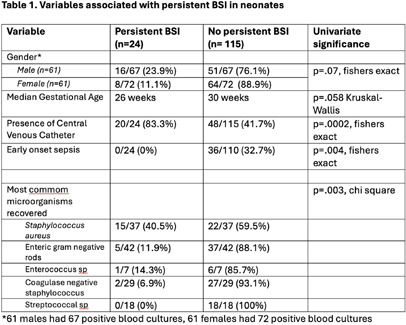# After an initial positive blood culture, when are repeat blood cultures necessary in the Neonatal Intensive Care Unit (NNICU)?

**DOI:** 10.1017/ash.2025.317

**Published:** 2025-09-24

**Authors:** Tom Murray, Hanna Lee, David Peaper, Matthew Bizzarro, Noa Fleiss

**Affiliations:** 1 Yale New Haven Children’s Hospital; 2 Yale University School of Medicine; 3 Yale University

## Abstract

**Background:** Data in adults and older children demonstrate repeat blood cultures (BlCx) are not always necessary. Indications for repeat BlCx include Staphylococcus aureus or yeast in the initial blood culture, or the presence of a central venous catheter (CVC). Blood collection in premature babies can be challenging and there are little data regarding when repeat BlCxs are necessary after an initial positive. The goal of this study is to determine risk factors for persistent bloodstream infection (BSI) to determine when unnecessary blood cultures can be avoided. **Methods:** The Yale New Haven Children’s Hospital NNICU is a 68-bed level 4 unit. Babies in the NNICU with a positive blood culture from 8/1/16 to 12/31/21 were included. Persistent BSI was defined as a repeat positive BlCx with the same organism >48 hrs. after the original culture. A BlCx > 7 days after the original BlCx was considered a new event. Babies who died within 48 hrs. of the initial culture were excluded. In preliminary analysis we did not distinguish between true BSI and contamination. Data were extracted from the medical record by the Yale Data Analytics Team and by manual chart review. Data were stored in excel for descriptive statistics. Additional statistical analysis in SPSS is on-going to account for multiple variables. **Results:** 142 babies had a positive BlCx with 122 babies alive at 48 hrs. and included in the study. These 124 babies had 139 positive BlCx growing 145 organisms. Persistent BSI occurred in 17.3% (24/139) of BlCxs. Factors associated with persistence in univariate analyses included the presence of a CVC and recovery of S. aureus. (Table 1) No babies with either streptococcal infection or early onset sepsis had persistent BSI. (Table 1) Additional variables under evaluation in a multiple regression model to determine the probability of persistent BSI include other sources of infection, white blood cell count at the time of BlCx, congenital heart disease, immunosuppressive agents such as steroids and whether empiric antibiotic therapy was appropriate. We will also define probable contaminants and repeat the analyses with and without these BSI episodes. **Conclusions:** Preliminary analysis shows that neonates have similar risk factors for persistent BSI as adults including the presence of a CVC and the recovery of S. aureus that require repeat BlCx to confirm clearance. For babies with streptococcal infection, repeat BlCx may not be routinely required. Current work is examining additional potential risk factors in multi-variable models.

**Figure:**